# Hospitals and Medical Schools

**Published:** 1904-11-12

**Authors:** 


### HOSPITALS AND MEDICAL SCHOOLS.

The Hon. Sydney Holland, writing to the *Morning Post*, says:—

"I suppose one ought to be very angry at having words such as "misappropriation of funds," "breach of sacred trust," and so on applied to any work with which one is connected, but anyone who knows Mr. Stephen Coleridge and his playful ways will estimate his abuse and his statements at their full value. I do not propose here to answer his characteristic letter, because the King's Fund have appointed an excellent committee to consider the question of hospitals paying money to their schools, and to decide whether they get a *quid pro quo* for these payments, and before that committee I hope to be allowed to give evidence. Meanwhile I will ask the public to believe that Mr. Stephen Coleridge is not the sole remaining man with any sense of honour left in this country, though I am quite sure he thinks he sees torpedoes."

